# The impacts of sex and the 5xFAD model of Alzheimer’s disease on the sleep and spatial learning responses to feeding time

**DOI:** 10.3389/fneur.2024.1430989

**Published:** 2024-07-31

**Authors:** Katrina J. Campbell, Peng Jiang, Christopher Olker, Xuanyi Lin, Sarah Y. Kim, Christopher J. Lee, Eun Joo Song, Fred W. Turek, Martha Hotz Vitaterna

**Affiliations:** ^1^Center for Sleep and Circadian Biology, Northwestern University, Evanston, IL, United States; ^2^Department of Neurobiology, Weinberg College of Arts and Sciences, Northwestern University, Evanston, IL, United States

**Keywords:** Alzheimer’s disease, time-restricted feeding, phase-restricted feeding, sleep, cognition, 5xFAD

## Abstract

**Introduction:**

The relationships between the feeding rhythm, sleep and cognition in Alzheimer’s disease (AD) are incompletely understood, but meal time could provide an easy-to-implement method of curtailing disease-associated disruptions in sleep and cognition. Furthermore, known sex differences in AD incidence could relate to sex differences in circadian rhythm/sleep/cognition interactions.

**Methods:**

The 5xFAD transgenic mouse model of AD and non-transgenic wild-type controls were studied. Both female and male mice were used. Food access was restricted each day to either the 12-h light phase (light-fed groups) or the 12-h dark phase (dark-fed groups). Sleep (electroencephalographic/electromyographic) recording and cognitive behavior measures were collected.

**Results:**

The 5xFAD genotype reduces NREM and REM as well as the number of sleep spindles. In wild-type mice, light-fed groups had disrupted vigilance state amounts, characteristics, and rhythms relative to dark-fed groups. These feeding time differences were reduced in 5xFAD mice. Sex modulates these effects. 5xFAD mice display poorer spatial memory that, in female mice, is curtailed by dark phase feeding. Similarly, female 5xFAD mice have decreased anxiety-associated behavior. These emotional and cognitive measures are correlated with REM amount.

**Discussion:**

Our study demonstrates that the timing of feeding can alter many aspects of wake, NREM and REM. Unexpectedly, 5xFAD mice are less sensitive to these feeding time effects. 5xFAD mice demonstrate deficits in cognition which are correlated with REM, suggesting that this circadian-timed aspect of sleep may link feeding time and cognition. Sex plays an important role in regulating the impact of feeding time on sleep and cognition in both wild-type and 5xFAD mice, with females showing a greater cognitive response to feeding time than males.

## 1 Introduction

Alzheimer’s disease (AD) is a highly prevalent neurodegenerative disease and is the most common cause of dementia (1); about 1 in 9 people 65 or older have AD, with about two thirds of these being women. In 2015, over 45 million people worldwide were estimated to have AD (1).

In AD, amyloid beta (Aβ) protein aggregates into plaques and hyperphosphorylated tau protein forms neurofibrillary tangles in the brain (2–7). There is a long preclinical stage in the development of AD during which protein pathology has begun but cognitive symptoms have not yet presented (8–12). Accompanying preclinical protein pathogenesis are disruptions in sleep and circadian rhythms (CRs) such as insomnia, increased daytime sleepiness, and sleep fragmentation that can appear years before a clinical diagnosis (13). These sleep disturbances are especially important due to the crucial role that sleep plays in memory (14, 15) and cognitive processes (16).

CRs describe the variations in behavior and physiology that cycle rhythmically at approximately 24 h in response to environmental and transcriptional cues. These include the sleep/wake cycle but there are also many other rhythms including: immune, cardiovascular, metabolic, and neurological rhythms. Circadian rhythms can be modulated directly by light exposure as well as indirectly by a number of other factors such as exercise, meal-times, and caffeine intake (17).

CRs are present in cells throughout the body and are critical for the coordination of molecular processes. The modulation of CRs by several environmental cues means that, when these cues are misaligned, CR misalignment can follow. The effects of circadian misalignment are obvious in shift work and jet lag where the endogenous circadian rhythms are misaligned with environmental cues. These have been linked to cancer risk, cardiometabolic disorders, motor dysfunction, neuroinflammation, and neurodegenerative diseases (18–34).

Just as circadian rhythms can be disrupted by mis-timed cues, they can be enhanced by properly placed cues. Feeding affects many of the cellular pathways associated with CRs, as well as the expression of circadian clock genes and by way of time-restricted feeding (TRF), these rhythms can be enhanced if the feeding occurs at an appropriate time (35–37). Research in this area suggests that the timing of the window is important and that mis-timed TRF in mice can induce obesity and metabolic disorders (35, 38). In wild-type mice, mis-timed TRF also inhibits hippocampal-dependent learning and memory (39).

The nature of the link between CR disruption and the development of AD is unclear (40–49), but it seems likely that the link is bidirectional. Insomnia and CR disruption increase the risk of developing later dementia (50, 51). Furthermore, sleep and CR disturbances increase the concentration and pathology of Aβ42 and tau in the nervous system, providing a direct link to the development of AD (45, 52–70). Contrariwise, Aβ and tau pathology as well as neurodegeneration can disrupt sleep and CRs (45, 71, 72). Importantly, genetic mutations in genes associated with Aβ tau used to recapitulate AD in model mice also confer sleep and circadian disturbances. APP23 and 5xFAD AD model mice both demonstrate reduced sleep as well as increased fragmentation (73–75).

An interesting extension of this paradigm is that enhancement of CRs directly by bright light stimulation reduces Aβ42 accumulation and cognitive impairment in 5xFAD mice (76). Furthermore, indirect enhancement of CRs by TRF has also been shown to be effective. In AD model mice, TRF without calorie restriction is able to improve sleep/activity rhythms and cognitive function as well as physiological markers of Aβ pathology and neurodegeneration (73, 77, 78).

In our study, we used the 5xFAD model of AD (79). The effects of TRF in 5xFAD mice, our AD model of interest have been established but the relative importance of the circadian timing of the food intake window has been less well-explored. Because our study was focused on the circadian phase of feeding rather than the effects of time-restriction of feeding, we refer here to our feeding protocol as phase-restricted feeding. The 5xFAD model is a very well-characterized and understood model. The sleep phenotype as well as the effectiveness of phase-restricted feeding has been described in this model and we wished to build on this. The 5xFAD model was also useful in that these mice experience rapid amyloid accumulation which causes neuronal loss leading to cognitive impairment (80, 81). We also used high-fat diet to capitalize on the metabolic aspects of AD. AD, sometimes referred to as type 3 diabetes, is correlated with metabolic function (82). In humans, diets high in saturated fat confer intake of dietary saturated and trans fats confer increased risk of AD and Mild Cognitive Impairment (83, 84). In fact, these fats enhance amyloid deposition and cognitive impairment in humans (85–87) and AD model mice (85, 86, 88). In 5xFAD mice, intracellular Aβ accumulates at 1.5 months (79), leading to extracellular Aβ deposition at 2 months (81) and neuronal loss at about 6 months (89, 90). In our study, the mice were about 3.5 months old when phase-restricted feeding began, 5.75 months at the time of EEG/EMG recording and 6 months at the time of the BMT. Combined with the HFD, it is likely that our model mice had significant plaque burden at the time of the experiment.

Taking into account the body of research on phase-restricted feeding and diet in the 5xFAD model of AD, we aimed to further investigate the effects of phase-restricted feeding as a circadian intervention which can modulate sleep, an established factor in both cognition and AD. As it has been well established that TRF can act as an effective zeitgeber, we wanted to explore if the length of the feeding window or the timing of the window is the crucial aspect of the intervention. Furthermore, we wanted to know if these effects are consistent across sex and genotype regarding wild-type and AD mice.

Our findings indicate that, in regards to sleep characteristics, AD model mice were less responsive to feeding time than WT mice and that females were more sensitive than males. These differences in REM sleep paralleled outcomes in memory.

## 2 Materials and methods

### 2.1 Animals

Transgenic mice which overexpress mutant human amyloid beta (A4) precursor protein 695 (APP) with the K670N, M671L, V717I familial Alzheimer’s disease (FAD) mutations and human presenilin 1 (PS1) with the M146L and L286V FAD mutations (5xFAD) heterozygous mice were bred in an on-site facility by crossing heterozygous 5xFAD males with wild-type (WT) B6SJLF1/J females, producing transgenic and WT offspring used in this study. 5xFAD genotypes were determined from PCR amplification of DNA from tail-tip biopsies collected at the time of weaning (66). Mice were screened for retinal degeneration (*Pde6b^rd1^*) and hypopigmented eyes (*Tyr^c^* or *Oca2^p^* mutations) (91); individuals homozygous for any of these mutations affecting light sensitivity were excluded from study. All mice were 3.5 months (± 1 week) old when phase-restricted feeding began.

### 2.2 Diet and housing

Mice were maintained on a 12:12 L:D (light intensity was approximately 500 lux) cycle at room temperature with water available *ad libitum*. The timing of procedures will be reported in zeitgeber time (ZT) in which ZT0 is lights on and ZT12 is lights off.

All mice were group-housed with regular chow diet (Envigo Teklad 7,912) until being separated into their individual home cages at the beginning of the study. High fat diet (HFD) mouse chow [60% kcal from fat; Research Diets Inc. (D12492)] was given to all mice when phase restricted feeding was initiated.

Three study cohorts totaling 49 mice completed the entire experiment within 2.5 months of each other. They were divided into 8 groups: 5xFAD light-fed female (*N* = 6), 5xFAD light-fed male (*N* = 6), 5xFAD dark-fed female (*N* = 8), 5xFAD dark-fed male (*N* = 7), wild-type light-fed female (*N* = 5), wild-type light-fed male (*N* = 7), wild-type dark-fed female (*N* = 4), wild-type dark-fed male (*N* = 6). In the legends, these are abbreviated as 5xFAD L (light-fed 5xFAD), 5xFAD D (dark-fed 5xFAD), WT L (light-fed wild-type), and WT D (dark-fed wild-type).

Mice were weighed at the beginning of the study before phase restricted feeding and again at weeks 3.5 and 6. The percent change in weight from baseline weight was calculated for each animal.

### 2.3 Phase restricted feeding

**Figure 1:**
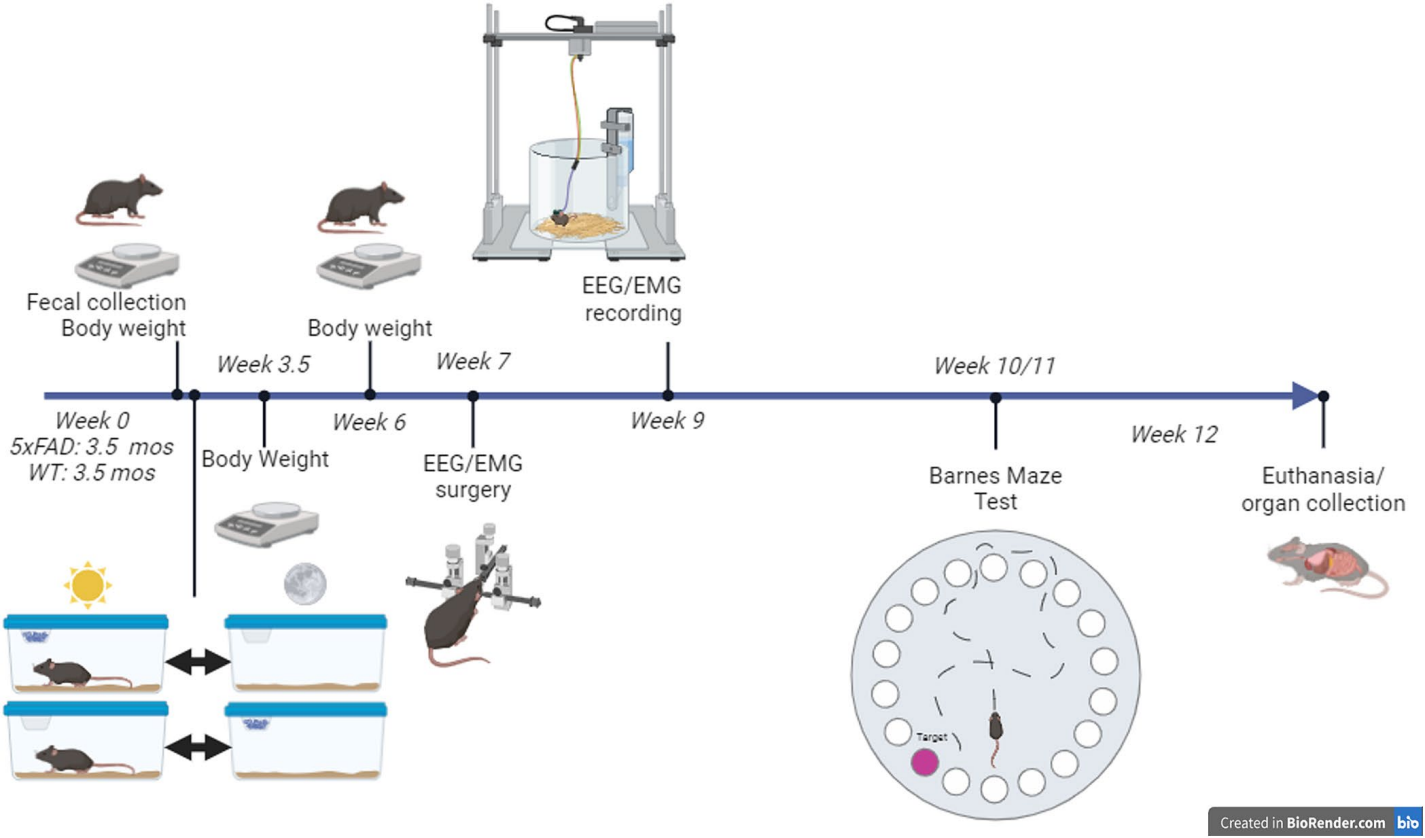
Summary of experimental protocol. Phase-restricted feeding started at 3.5 months of age. All mice were provided with two “home” cages: one for the 12-h light phase and one for the 12-h dark phase that they were transferred between at lights-on and lights-off each day. High-fat diet was provided in one of the cages, depending on whether the mouse was assigned to the light-fed or dark-fed treatment group.

All mice had access to HFD via a top-of-cage food hopper either during the 12 h light phase, the “incorrect phase” or the 12 h dark phase, the “correct phase” each day. The phase-restricted feeding was achieved using 2 home cages to which the mice were well-acclimated; one for the light phase and one for the dark phase. Each mouse was transferred between the cages twice every day within half an hour of lights-on and of lights-off (35) (Figure 1). Mice were acclimated to the cage-change protocol for 2 days before phase-restricted feeding began and for 3 days before the EEG/EMG recording. The cage change protocol did not increase the amount of time for mice to enter NREM when compared to mice fed either RD or HFD with no switch protocol (Supplementary Figure 1).

### 2.4 Sleep recording

When mice were approximately 5–5.5 months old, 7 weeks after phase-restricted feeding began (Figure 1), surgery was performed on all mice to implant an electroencephalographic/electromyographic (EEG/EMG) sleep recording device (Pinnacle Technologies). See Bowers et al. (92) for details (only the anesthesia method differed). Briefly, surgery was performed on a stereotaxic apparatus. Anesthesia consisted of Isoflurane, USP (Covetrus) carried by medical grade oxygen (Airgas) with the RC2 rodent circuit controller (VetEquip) beginning at a range of 1.5%–2% and adjusted as needed to maintain a surgical plane of anesthesia. Each headmount (Pinnacle Technology Inc.) was secured to the skull with dental acrylic. Mice were given a dose of the analgesic meloxicam (2 mg/kg; Norbrook Laboratories) at the time of surgery and the day after surgery.

One week after surgery, mice were moved into the sleep recording chambers and connected to the sleep recording equipment (Figure 1). Each sleep recording chamber housed one mouse with two cages (one for the light phase, one for the dark). Cages had bedding and water available *ad libitum*. Mice were immediately connected to a 4-channel tethered system (Pinnacle Technology Inc.) via a preamplifier. After a 2 day acclimation phase, EEG/EMG data were collected with the Pinnacle Acquisition software (Pinnacle Technology Inc.) over 48 h.

### 2.5 Sleep measures analysis

A machine-learning based sleep scoring system developed in the laboratory was used to score vigilance states, i.e., 10-s epochs were classified as wake, non-REM sleep (NREM) or Rapid Eye Movement sleep (REM) based on “learned” classifications from expert human scoring of approx. 8% of each individual data file. EEG frequency bands were identified as delta (0.5–4 Hz), theta (4–8 Hz), alpha (8–11 Hz), sigma (11–15 Hz) and beta (15–30 Hz). Sleep parameters were calculated from each scored file (93), including vigilance state amounts, number and duration of state bouts, state changes and power of frequency by vigilance state. For analyses, these parameters were evaluated in either 3, 12, or 24 h bins. For three-hour bins, bins are labeled with the initial time.

To evaluate rhythmic aspects of sleep, time of day was treated as a circular (rather than linear) scale. The mean vector provided descriptive statistics of the central tendency of vigilance state rhythms (94). The mean angle indicated peak time, while the vector length reflected dispersion/concentration. Vector length ranges from 0 (high dispersion/low amplitude) to 1 (high concentration/high amplitude) and was used as a measure of relative rhythm amplitude.

Sleep spindles and their characteristics were calculated based on previously published methods using modifications (95). In our analysis for this paper, we used standard deviation (+0.5 SD primary threshold +2.0 SD secondary threshold) to define spindles. We also used an 800 ms window that moves by 200 ms due to our standard EEG epoch length and sampling rate (250 Hz).

### 2.6 Barnes maze test

10 weeks after phase-restricted feeding was begun, mice underwent Barnes Maze test (BMT; Figure 1). The BMT consisted of one habituation session on day 1, two trials per day on days 1–5, and one probe test on day 12. Procedures began at approximately ZT1.

The maze consisted of a polyvinyl chloride foam board circle 47 inches in diameter with 36 holes. The escape tunnel, an opaque laminate box lined with a disposable task wipe, was placed underneath the escape hole for all trials. For habituation, this escape tunnel was placed under an alternate hole. The start box was an opaque plastic beaker. The maze room included many orientation objects and was lit with a dim light. White noise was played to drown out extraneous noise. The test was recorded by a camera placed directly above the maze. All surfaces the mouse interacted with were cleaned with ethanol between trials. The mice were stored in an adjacent room between tests.

During the habituation session, the mouse was brought into the room via the escape tunnel and placed under a covered hole for 1 min. The mouse was moved to the maze surface and allowed to explore for 5 min. If the mouse entered the escape tunnel, the test was concluded. If, at the end of 5 min, it had not, the mouse was guided to and placed in the tunnel. After 15 s in the escape tunnel under a covered hole, the mouse was removed.

For the trials, the escape tunnel was placed under the escape hole. The mouse was brought into the room using the covered start box and placed in the center of the maze for 15 s. The start box was then removed. The mouse was given 3 min to find the escape tunnel. If the mouse entered the escape tunnel, the trial was concluded. If the mouse did not enter the escape tunnel, it was guided to and placed in the tunnel. After 15 s under the covered escape hole, the mouse was removed. All of the mice from each cohort underwent trial one and then trial two so the time between trials for each mouse was approximately 2 h.

For the probe test, the escape tunnel was not attached to the maze apparatus. The mouse was brought into the room in the covered start box and placed in the center of the maze for 15 s after which the start box was removed. The animal was allowed to explore for 1 min.

Scored behaviors were used to evaluate spatial learning and memory. These including: time to first investigate the escape hole (primary latency) and the number of incorrect holes investigated before the escape hole (primary errors). From the Barnes maze videos, we also scored the amount of time spent in the center of testing table as opposed to the edges as well as freezing (movement hesitation) as a measure of anxiety. These cognitive and behavioral measures were correlated with sleep measures.

### 2.7 Statistical analyses

Sleep and behavioral measures were analyzed by 2- or 3-way Analysis of Variance (ANOVA) to examine effects of sex, genotype, or feeding time as well as interactions among two or all of these factors. Student’s t-test was used for post-hoc pairwise comparisons where appropriate. Relationships between sleep and behavioral phenotypes were evaluated using Pearson’s correlation with Bonferroni correction for multiple comparisons.

## 3 Results

### 3.1 Body weight changes

**Figure 2:**
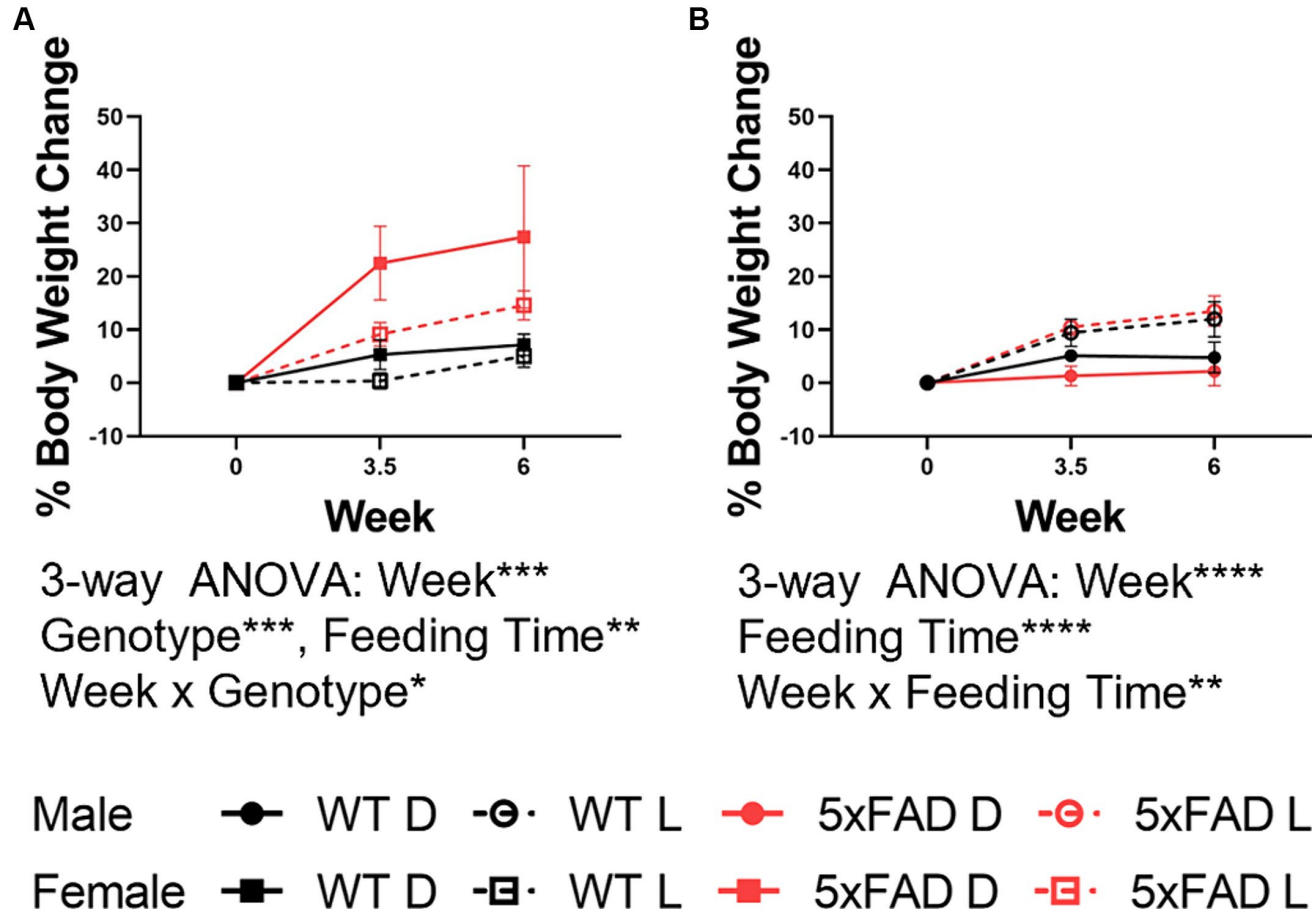
Body weight changes in WT and 5xFAD mice. Percent change from baseline in body weight in female **(A)** and male **(B)** WT and 5xFAD mice through the first 6 weeks of the experiment. Data are presented as mean ± SEM. Statistical significance was determined by 3-way ANOVA (genotype × feeding time × week). ^*^p < 0.05; ^**^p < 0.01; ^***^p < 0.001; ^****^p < 0.0001. *N* = 3–7 mice per group.

All animals gained weight over the course of the study despite phase-restricted access to food. Changes in body weight were sex-dependent. In males (Figure 2B), WT and 5xFAD mice both gained equivalent weight over the first 6 weeks of the experiment and in both genotypes, light-fed mice gained more weight than dark-fed mice (3-way ANOVA *p* = 0.0001). In females (Figure 2A), 5xFAD mice gained more weight than WT mice (3-way ANOVA *p* = 0.0007) and, contrary to the males, gained more weight when fed during the dark phase (3-way ANOVA *p* = 0.0089). Changes in body weight were not associated with changes in sleep amount (Supplementary Figure 2).

### 3.2 Impacts of feeding time on wake and NREM in WT and 5xFAD mice

**Figure 3:**
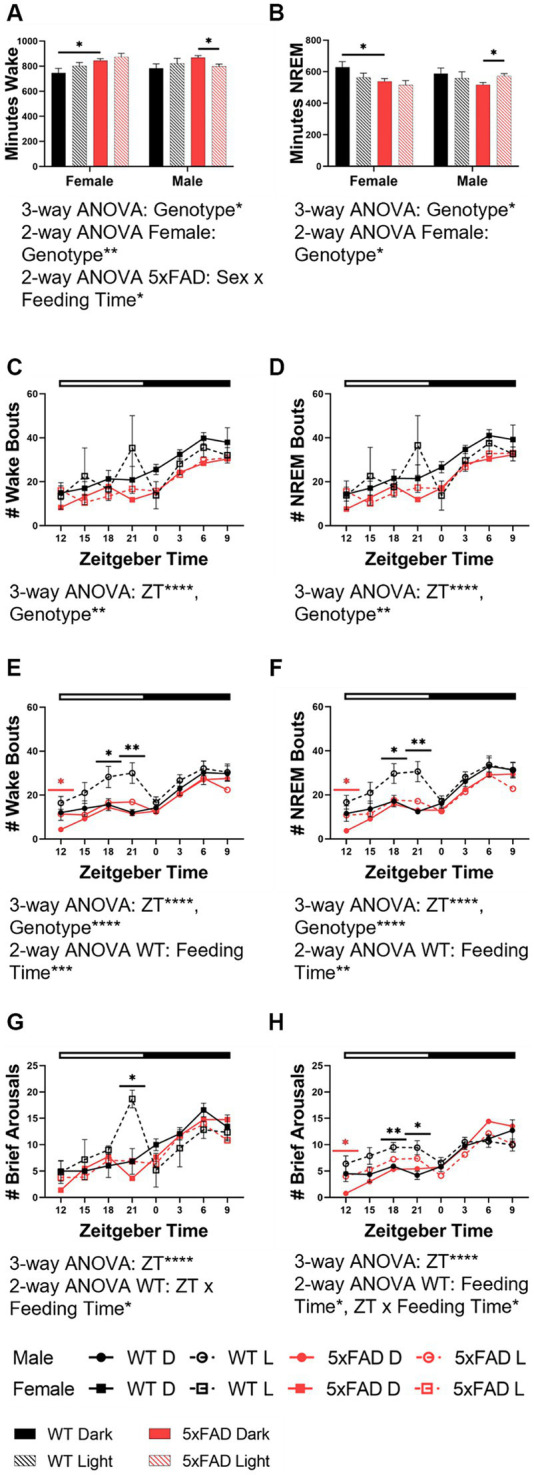
Wake and NREM characteristics in WT and 5xFAD mice. The total time in minutes over 24 h which was spent in wake **(A)** and NREM **(B)** in all experimental groups. Number of total wake and NREM bouts across 24 h in female (wake **C**, NREM **D**) and male (wake **E**, NREM **F**) WT and 5xFAD mice. Number of brief arousals across 24 h in female **(G)** and male **(H)** WT and 5xFAD mice. Data are presented as mean ± SEM. Statistical significance was determined by 3-way ANOVA [genotype × feeding time × sex **(A,B)**, genotype × ZT × feeding time **(C–H)**], 2-way ANOVA [genotype × feeding time **(A,B)**, sex × feeding time **(A,B)**, ZT × feeding time **(C–H)**], and two-tailed two-sample Student’s t-test. ^*^p < 0.05; ^**^p < 0.01; ^***^p < 0.001; ^****^p < 0.0001. In panels **(C–H)**, black asterisks indicate differences between WT mice, red asterisks indicate differences between 5xFAD mice. *N* = 3–7 mice per group.

The amount and characteristics of wake and NREM were modulated by feeding time, genotype, and sex. We first measured the amount of time spent in wake (Figure 3A) and NREM (Figure 3B) as a broad measure of diurnal rhythms. The amount of wake and NREM were disrupted both by the 5xFAD genotype and mistimed feeding. The nature of these disruptions were influenced by sex. 5xFAD mice spent an increased amount of time in wake (3-way ANOVA *p* = 0.015) and decreased time in NREM (3-way ANOVA *p* = 0.0034) in both female and male mice. In WT mice, feeding during the light phase consistently increased wake in both females and males, although to a nonsignificant level. In 5xFAD mice; however, sex interacted with feeding time to influence wake (2-way ANOVA *p* = 0.033). In female mice, both the WT and 5xFAD genotypes increased wake in response to light-phase feeding (2-way ANOVA *p* = 0.0089) while in male 5xFAD mice, wake decreased slightly in response to light-phase feeding.

Beyond the overall amount of wake and NREM, we also investigated the properties of these vigilance states. Fragmentation was the next measure we considered. We calculated fragmentation over the course of the 24 h period in 3 h bins. The number of wake [females (Figure 3C), males (Figure 3E)] and NREM [females (Figure 3D), males (Figure 3F)] bouts varied over time (3-way ANOVA *p* < 0.0001 all groups) and were influenced by genotype in all groups (female wake, NREM *p* = 0.001, 0.002; male wake, NREM, *p* < 0.001, 0.001). In WT female and male mice, an increase in the number of wake and NREM bouts at the end of the dark phase was apparent in light-fed mice when compared to dark-fed, although the divergence was seen more throughout the dark phase in males. The number of bouts in 5xFAD mice was less dependent on feeding time. In female 5xFAD mice, the number of bouts was consistent, regardless of feeding time. Male 5xFAD mice were much more consistent than WT counterparts but a small increase in the number of bouts was seen at the beginning of the dark phase in wake and NREM. A similar pattern in was seen in the number of brief arousals in which feeding time impacted the number of brief arousals in female (Figure 3G) and male (Figure 3H) WT (2-way ANOVA *p* = 0.012, 0.012) but not 5xFAD mice.

### 3.3 Effects of feeding time on the rhythm characteristics of wake and NREM in WT and 5xFAD mice

**Figure 4:**
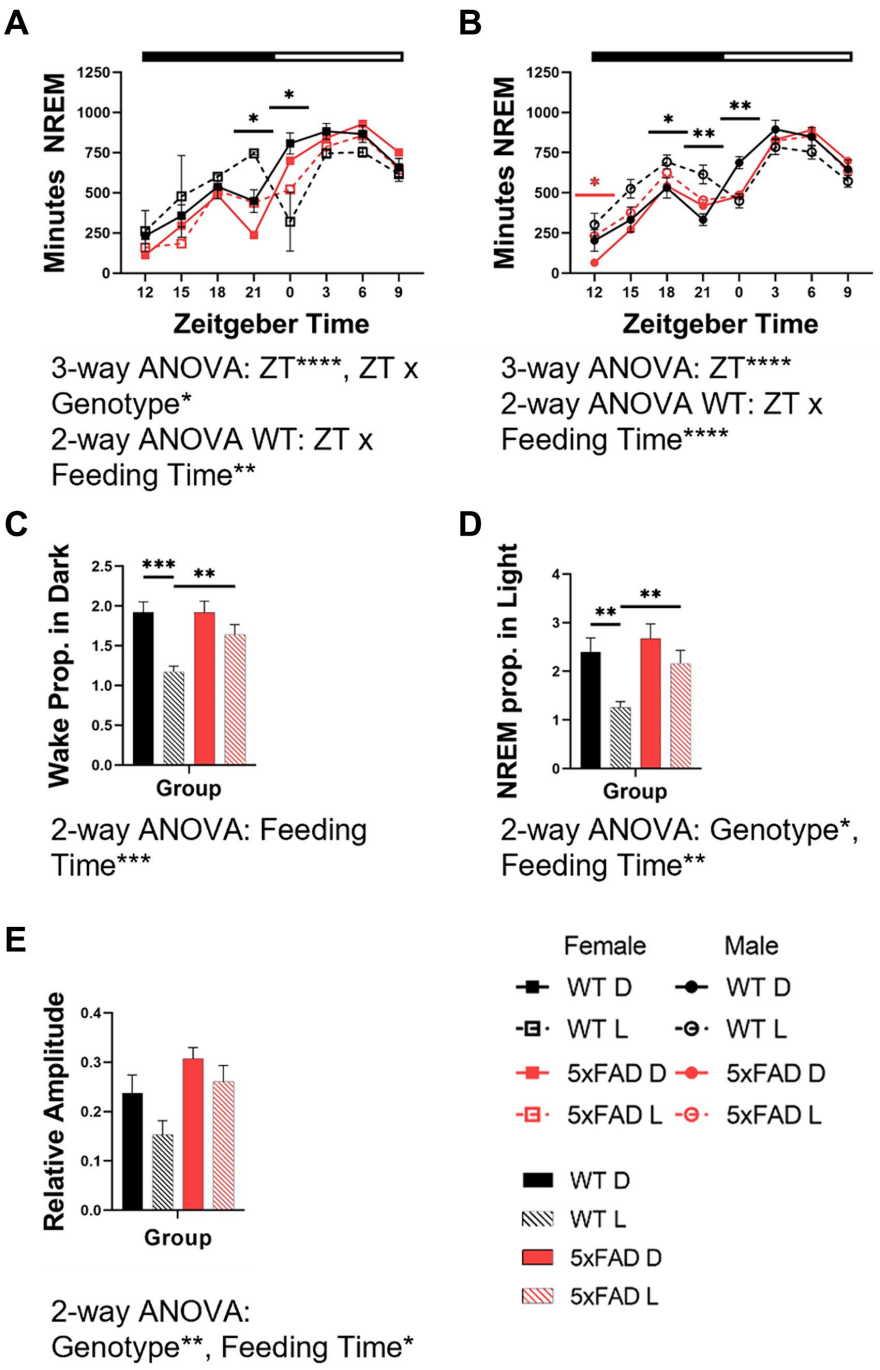
Effects of feeding time on the rhythm characteristics of wake and NREM in WT and 5xFAD mice. NREM across 24 h in female **(A)** and male **(B)** mice. Proportion of wake **(C)** and NREM **(D)** which occur in the expected phase for nocturnal mice (wake in dark phase, NREM in light phase) in female and male WT and 5xFAD mice. Relative amplitude as a representation of phase consolidation of NREM **(E)**. Data are presented as mean ± SEM. Statistical significance was determined by 3-way ANOVA [genotype × feeding time × ZT **(A,B)**], 2-way ANOVA [ZT × feeding time **(A,B)**, genotype × feeding time **(C,D,E)**], and two-tailed two-sample Student’s t-test. ^*^p < 0.05; ^**^p < 0.01; ^***^p < 0.001; ^****^p < 0.0001. In panels **(A,B)**, black asterisks indicate differences between WT mice. *N* = 3–7 mice per group.

The diurnal rhythms of wake (not shown) and NREM in both female and male mice were disrupted by light-time feeding. We calculated the amount of time spent in NREM over 48 h in 3-h bins in WT and 5xFAD mice. Both female (Figure 4A) and male (Figure 4B) WT mice fed during the dark phase had appropriately-timed NREM which was disrupted by light-time feeding (3-way ANOVA *p* = 0.0004, 0.0003). In all WT mice fed during the light-time, NREM was transposed from the beginning of the light phase to the end of the dark phase. In female 5xFAD mice, NREM maintained this pattern of transposition but to a lesser degree than was seen in WT mice. In male 5xFAD mice, this transposition did not occur, and the effect of the diet timing was seen instead in the beginning of the dark with increased NREM.

Interestingly, the 5xFAD genotype caused a consolidation of vigilance states into the correct phase due to decreased wake in the light and sleep in the dark phases. This resulted in an increased proportion of wake (Figure 4C) and NREM (Figure 4D) in the correct phase (2-way ANOVA NREM *p* = 0.0001, 0.029). The proportion of wake and NREM which occurred during the correct phase (wake in dark, NREM in light) decreased with mistimed feeding (3-way ANOVA *p* = 0.0001, 0.0001).

The amplitude of wake (not shown) and NREM rhythms were disrupted by light-time feeding. The 5xFAD genotype resulted in an increased consolidation of NREM (3-way ANOVA *p* = 0.014). In all genotypes and sexes, NREM consolidation was decreased by light-time feeding (3-way ANOVA *p* = 0.047; Figure 4E).

### 3.4 Effects of phase-restricted feeding on REM in WT and 5xFAD mice

**Figure 5:**
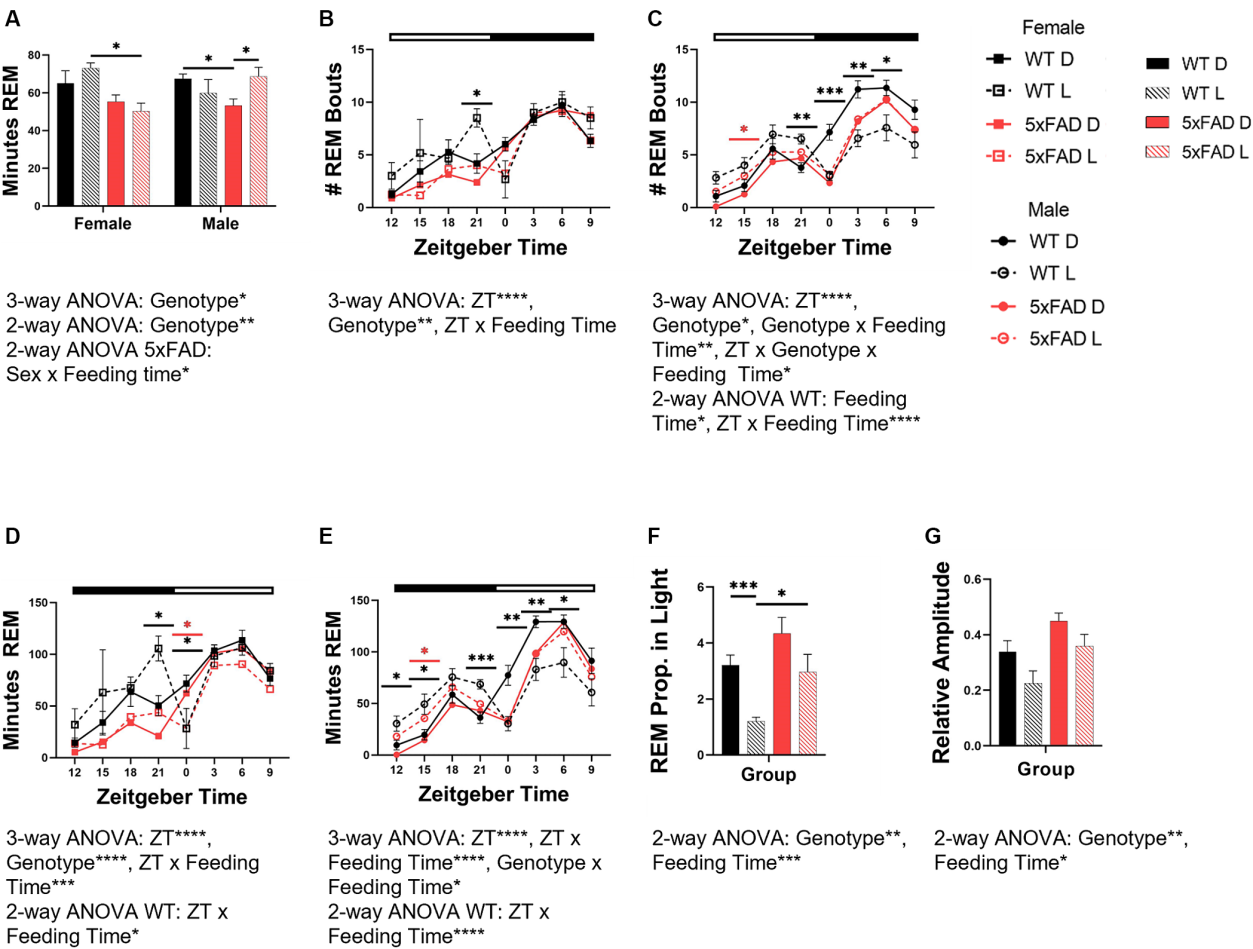
Effects of phase-restricted feeding on REM in WT and 5xFAD mice. The total number of minutes spent in REM over 24 h which was spent in REM was calculated for all experimental groups **(A)**. Number of total REM bouts in female **(B)** and male **(C)** mice. Minutes of REM across the day in female **(D)** and male **(E)** WT and 5xFAD mice. The proportion of REM which occurs in the correct phase (light phase) in WT and 5xFAD mice **(F)**. Relative amplitude as a representation of phase consolidation of REM **(G)**. Data are presented as mean ± SEM. Statistical significance was determined by 3-way ANOVA [genotype × feeding time × sex **(A)**, genotype × ZT × feeding time **(B–E)**], 2-way ANOVA [genotype × feeding time **(A,F,G)**, sex × feeding time **(A)**, ZT × feeding time **(B–E)**] and two-tailed two-sample Student’s t-test. ^*^p < 0.05; ^**^p < 0.01; ^***^p < 0.001; ^****^p < 0.0001. In panels **(B–E)**, black asterisks indicate differences between WT mice, red asterisks indicate differences between 5xFAD mice. *N* = 3–7 mice per group.

The 5xFAD genotype influenced REM (3-way ANOVA *p* = 0.012; Figure 5A) and interacted with sex to modulate REM amount (3-way ANOVA *p* = 0.017). In females but not males, 5xFAD mice had less REM (2-way ANOVA *p* = 0.0043). In males light-restricted feeding caused WT mice to decrease REM but 5xFAD mice to increase REM (2-way ANOVA *p* = 0.032).

The number of bouts for REM varied over the course of the day and was influenced by genotype in all groups [females (Figure 5B), males (Figure 5C)] (3-way ANOVA time *p* < 0.0001 all groups; genotype: female REM *p* = 0.005; male REM *p* = 0.013). In WT, but not 5xFAD female and male mice, an increase in the number of REM bouts at the end of the dark phase was apparent in light-fed mice when compared to dark-fed. In female 5xFAD mice, the number of bouts throughout the day was consistent, regardless of feeding time. In male mice, the divergence was seen more throughout the dark phase than was observed in female mice. In REM specifically, the feeding time influenced the number of bouts, and interacted with ZT (2-way ANOVA *p* = 0.013, < 0.0001). In light-fed animals, the number of REM bouts was lower during the light and higher during the dark when compared to dark-fed mice.

The rhythm of REM amount in both female (Figure 5D) and male (Figure 5E) mice was disrupted by light-time feeding. Both female and male WT mice fed during the dark phase had appropriately-timed REM which varied over time (all 3-way ANOVA *p* < 0.0001). The timing of REM in female and male WT mice was sensitive to feeding time, and became disrupted with light-time feeding (3-way ANOVA *p* = 0.0004, <0.0001). In female WT mice fed during the light phase, REM was transposed from the beginning of the light phase to the end of the dark. REM in light-fed male WT mice was increased throughout the entirety of the dark phase and decreased throughout the light. REM disruption was less severe in 5xFAD mice. In female 5xFAD mice, REM maintained the pattern seen in WT mice but to much less of a degree than seen in WT mice. In male 5xFAD mice, this transposition did not occur, and the effect of the diet timing was seen instead in the beginning of the dark with increased NREM and REM.

The 5xFAD genotype induced an increased proportion of REM in the light phase (3-way ANOVA REM *p* = 0.0027). Feeding mice in all groups during the light phase decreased the proportion of REM in the light phase (3-way ANOVA *p* = 0.0006; Figure 5F).

The 5xFAD genotype resulted in an increased consolidation of REM (3-way ANOVA *p* = 0.0047) due to decreased REM in the dark phase (Figure 5G). In all genotypes and sexes, REM consolidation is decreased by light-time feeding (3-way ANOVA *p* = 0.017).

### 3.5 Relative power spectral density analysis in WT and 5xFAD mice

Following our exploration of vigilance state amounts and diurnal characteristics, we examined the EEG signatures of the relative power spectral density (PSD) and sleep spindles.

**Figure 6:**
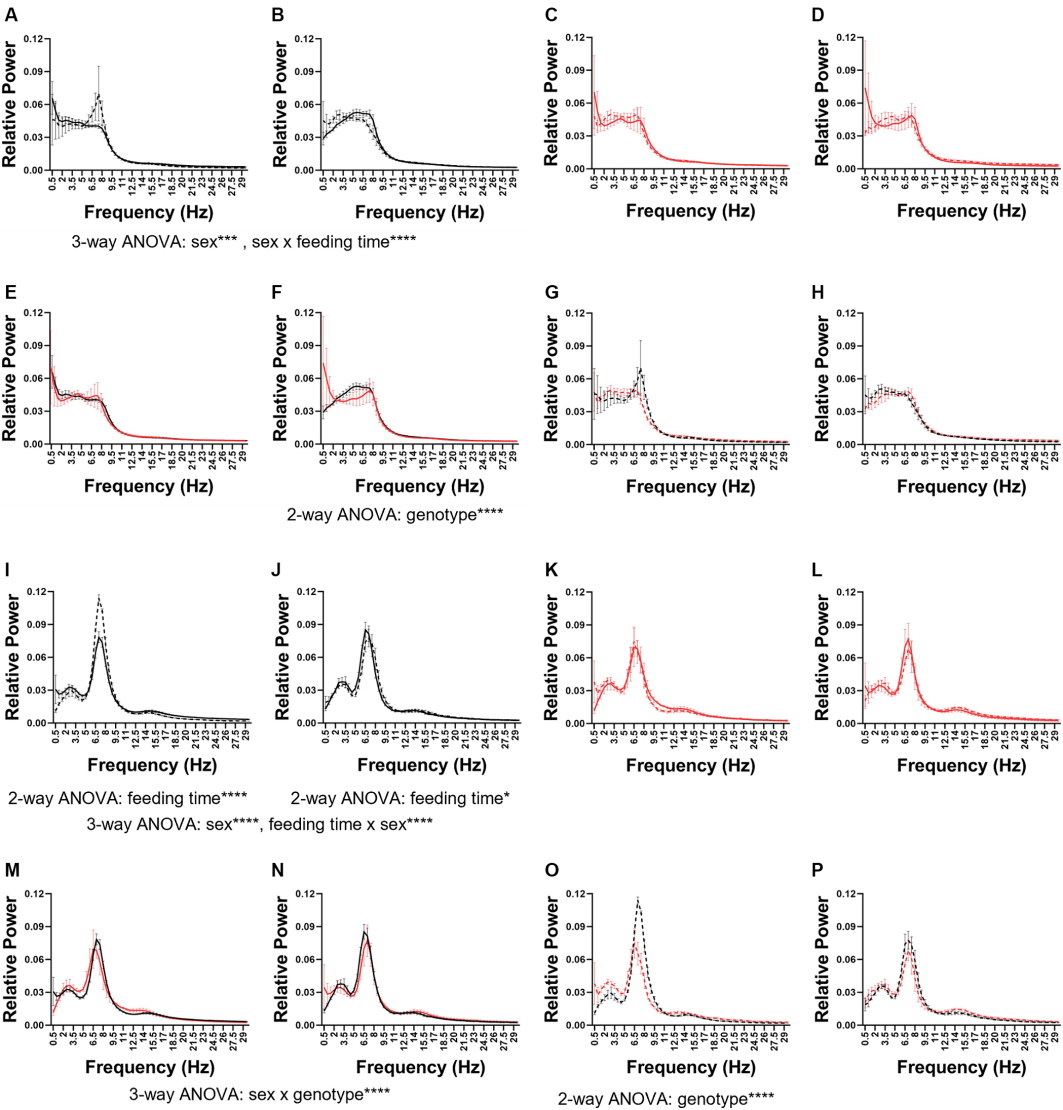
Wake and REM power spectral density. The wake **(A–H)** and REM **(I–P)** power spectral density in female **(A,C,E,G,I,K,M,O)** and male **(B,D,F,H,J,K,L,N,P)** mice. The PSDs were combined first by genotype: WT **(A,B,I,J)** and 5xFAD **(E,F,M,N)** and then by feeding time: dark **(C,D,K,L)** and light **(G,H,O,P)**. Data are presented as mean ± SEM. Statistical significance was determined by 2-way ANOVA (frequency × feeding time; frequency × genotype) and 3-way ANOVA (sex × genotype × frequency; feeding time × genotype × frequency). ^*^p < 0.05; ^***^p < 0.001; ^****^p < 0.0001. *N* = 3–7 mice per group.

As seen in previous sections 5xFAD were immutable compared to WT mice. In WT mice, wake PSD was affected by sex (3-way ANOVA sex × feeding time × genotype *p* = 0.0004) and feeding time interacted with sex (3-way ANOVA sex × feeding time × genotype *p* < 0.0001; Figures 6A,B). These changes occurred delta to alpha (0.5–11 Hz) range. In 5xFAD mice, no differences due to sex or feeding time were observed (Figures 6C,D).

In dark-fed mice, the 5xFAD genotype modified wake PSD in male (Figure 6F) but not female (Figure 6E) mice (2-way frequency × feeding time ANOVA *p* < 0.0001), specifically in the theta range. No significant differences were seen between light-fed WT and 5xFAD mice (Figures 6G,H). In wake, the PSD in males was more sensitive to genotype and feeding time (3-way ANOVA frequency × genotype × feeding time *p* = 0.0002) than females.

In both WT females (Figure 6I) and males (Figure 6J), feeding time affected REM PSD (2-way ANOVA feeding time × frequency WT females: *p* < 0.0001; WT males *p* = 0.0456). In females, this was due to a large increase in theta and in males a much more subtle shift in theta and alpha. Sex influenced REM PSD independently and interacted with feeding time (3-way ANOVA sex × feeding time × frequency sex: *p* < 0.0001; feeding time × sex *p* < 0.0001). Neither sex nor feeding time modified REM PSD in 5xFAD mice (Figures 6K,L). In dark-fed mice, sex and genotype interacted to influence REM PSD (3-way ANOVA sex × genotype × frequency *p* < 0.0001; Figures 6M,N). In light-fed mice, genotype only influenced REM PSD in females (2-way ANOVA genotype × frequency *p* < 0.0001; Figures 6O,P). Genotype, feeding time, and frequency influenced the PSD of REM in female mice (3-way ANOVA frequency × genotype × feeding time *p* < 0.0001) but in male mice, only genotype influenced REM PSD (3-way ANOVA frequency × genotype × feeding time *p* = 0.0026).

In WT mice, the PSD of NREM varied by sex (3-way ANOVA frequency × sex × feeding time *p* < 0.0001) but not feeding time (Supplementary Figures 3A,B). In female but not male 5xFAD mice, NREM PSD does vary by feeding time (2-way ANOVA frequency × feeding time *p* = 0.0005; Supplementary Figures 3C,D). The PSD of NREM varied by genotype in dark-fed (Supplementary Figures 3E,F; 2-way ANOVA frequency × genotype *p* = 0.0037) as well as light-fed (Supplementary Figures 3G,H; 2-way ANOVA frequency × genotype *p* = 0.0081) females but not significantly so in males. Sex, genotype, and frequency interacted to influence NREM PSD in both dark-fed (3-way ANOVA sex × genotype × frequency *p* < 0.0001) and light-fed mice (3-way ANOVA frequency × sex × genotype *p* = 0.0021). Overall, phenotype, feeding time, and frequency influenced the PSD of NREM in females (3-way ANOVA frequency × genotype × feeding time *p* < 0.0001) whereas in males, NREM PSD is influenced genotype (3-way ANOVA frequency × genotype × feeding time *p* = 0.0040).

### 3.6 Characterization of sleep spindles in WT and 5xFAD mice

**Figure 7:**
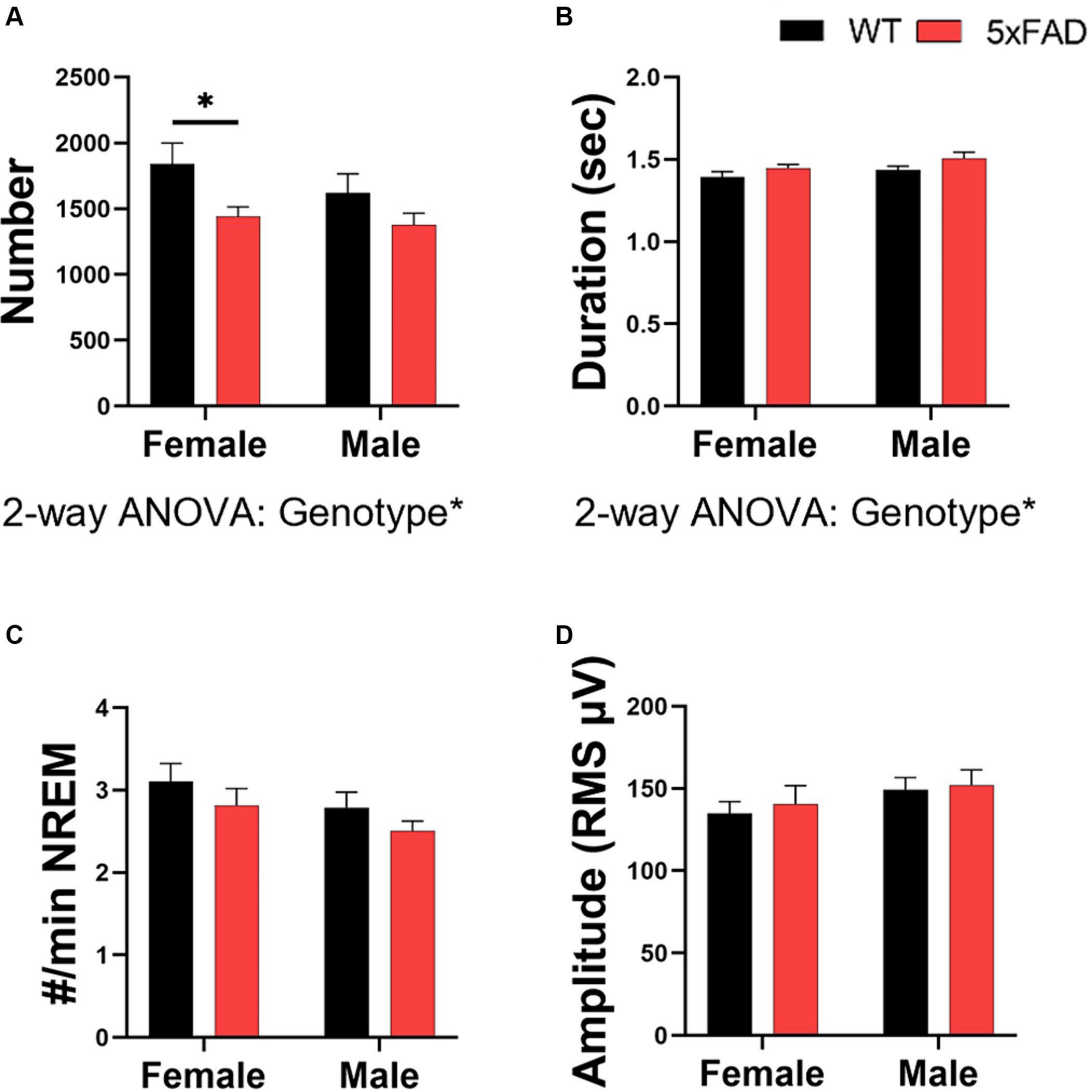
Characterization of sleep spindles in WT and 5xFAD mice. The number of spindles over 24 h **(A)** and the average duration of each spindle **(B)**. The number of spindles per minute of NREM **(C)**. Spindle amplitude as calculated by the root mean square **(D)**. Data are presented as mean ± SEM. Statistical significance was determined by 3-way ANOVA [genotype × feeding time × sex **(A–D)**], 2-way ANOVA [Genotype × sex **(A–D)**] and two-tailed two-sample Student’s t-test. ^*^p < 0.05; ^**^p < 0.01; ^***^p < 0.001. *N* = 3–7 mice per group.

5xFAD mice have small deviations in sleep spindles when compared to WT mice. 5xFAD mice have fewer (2-way ANOVA *p* = 0.027; Figure 7A) but slightly longer (2-way ANOVA *p* = 0.043; Figure 7B) sleep spindles than WT mice. No differences in spindle density (Figure 7C) or amplitude (Figure 7D) were found in any of the factors we investigated. There were no differences due to sex or feeding time in spindle number, duration, density, or amplitude.

### 3.7 Spatial learning in WT and 5xFAD mice

**Figure 8:**
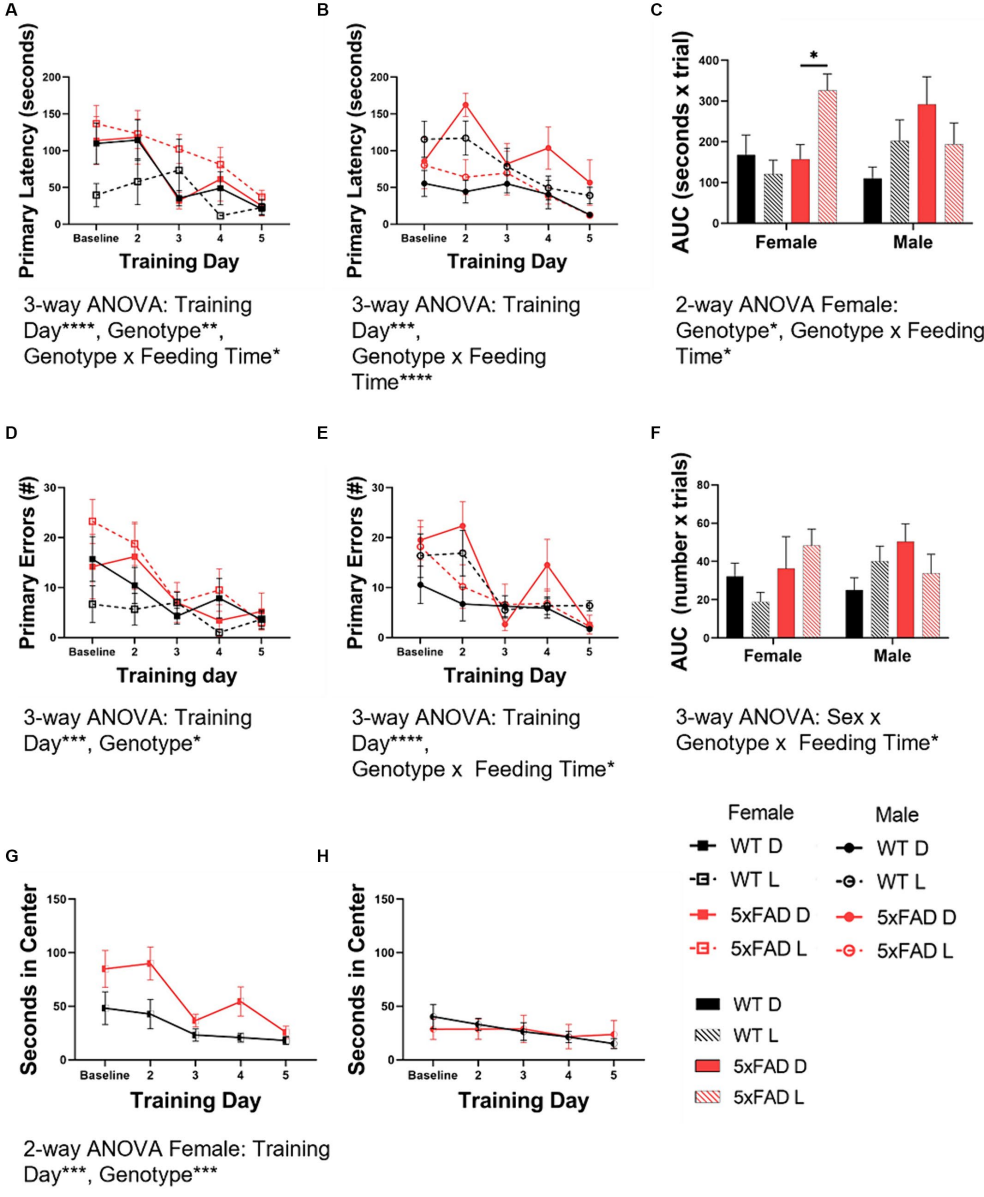
Spatial learning in WT and 5xFAD mice. Primary latency [female **(A)**, male **(B)**] and primary errors [female **(D)**, male **(E)**] in the Barnes maze test were calculated at baseline and throughout the testing days. These data were used to calculate the area under the curve (AUC) for both primary latency **(C)** and primary errors **(F)** as a representation of overall learning. The amount of time in seconds female and male mice spent in the center of the Barnes maze test table were calculated at baseline and throughout the testing days for female **(G)** and male **(H)** mice. Bar graph data are presented as mean ± SEM. Correlation data are presented as individual values. Statistical significance was determined by 3-way ANOVA [genotype × feeding time × training day **(A–F)**], 2-way ANOVA [genotype × training day **(G,H)**], and two-tailed two-sample Student’s t-test **(A–C)**. ^*^p < 0.05; ^**^p < 0.01; ^***^p < 0.001; ^****^p < 0.0001. *N* = 3–7 mice per group.

We utilized the Barnes maze test to asses spatial learning. We used the primary latency and primary errors as representations of learning in all mouse groups. Primary latency data indicated that both WT and 5xFAD mice learned over the course of the test (3-way ANOVA female, male *p* < 0.0001, =0.0004). Similar to the vigilance state data, genotype interacted with feeding time in influencing primary latency in both females (Figure 8A) and males (Figure 8B; 3-way ANOVA *p* = 0.026, 0.0004). Along with the time series data, we calculated the area under the curve (Figure 8C) as a representation of overall primary latency and learning. In females, 5xFAD mice had a longer primary latency (2-way ANOVA *p* = 0.0096) and light-fed 5xFAD mice had the longest latency (2-way ANOVA *p* = 0.0006). In males, WT mice fed during the light had a longer primary latency than those fed in the dark (*p* = 0.0017). The data from primary errors was consistent with the primary latency data. Both WT and 5xFAD mice had fewer errors as the test progressed (3-way ANOVA female, male *p* = 0.0006, < 0.0001). In females (Figure 8D), 5xFAD mice made more errors (3-way ANOVA *p* = 0.030). In males (Figure 8E), feeding time interacted with genotype (3-way ANOVA *p* = 0.011); light-fed WT mice made more errors (2-way ANOVA *p* = 0.005). There was no difference due to feeding time in 5xFAD mice. The area under the curve (Figure 8F) of the primary errors was calculated to compare the total errors made over the course of the test. In this measure, sex, genotype, and feeding time interacted to influence the number or primary errors (3-way ANOVA *p* = 0.044). The primary latency and primary errors of the Barnes maze test training period do not correlate with the primary latency and primary errors observed during the probe test (Supplementary Figure 4).

During the course of the BMT, we noted that some of the mice spent an inordinate amount of time in the center of the testing table. As time spent exposed in an open environment indicates a lack of anxiety, this time was scored as an inverse measure of anxiety. Female mice (Figure 8G) spent less time in the center with exposure (2-way ANOVA *p* = 0.0002) and female 5xFAD mice spent more time in the center than WT mice (2-way ANOVA *p* = 0.0003). Male mice (Figure 8H) spent minimal time in the center of the table throughout the entirety of the test, without variation over time. Freezing behavior (initial hesitation to move) was quantified separately but did not contribute significantly to the amount of time spent in the center of the testing table (data not shown).

### 3.8 Correlation between sleep and cognitive measures

**Figure 9:**
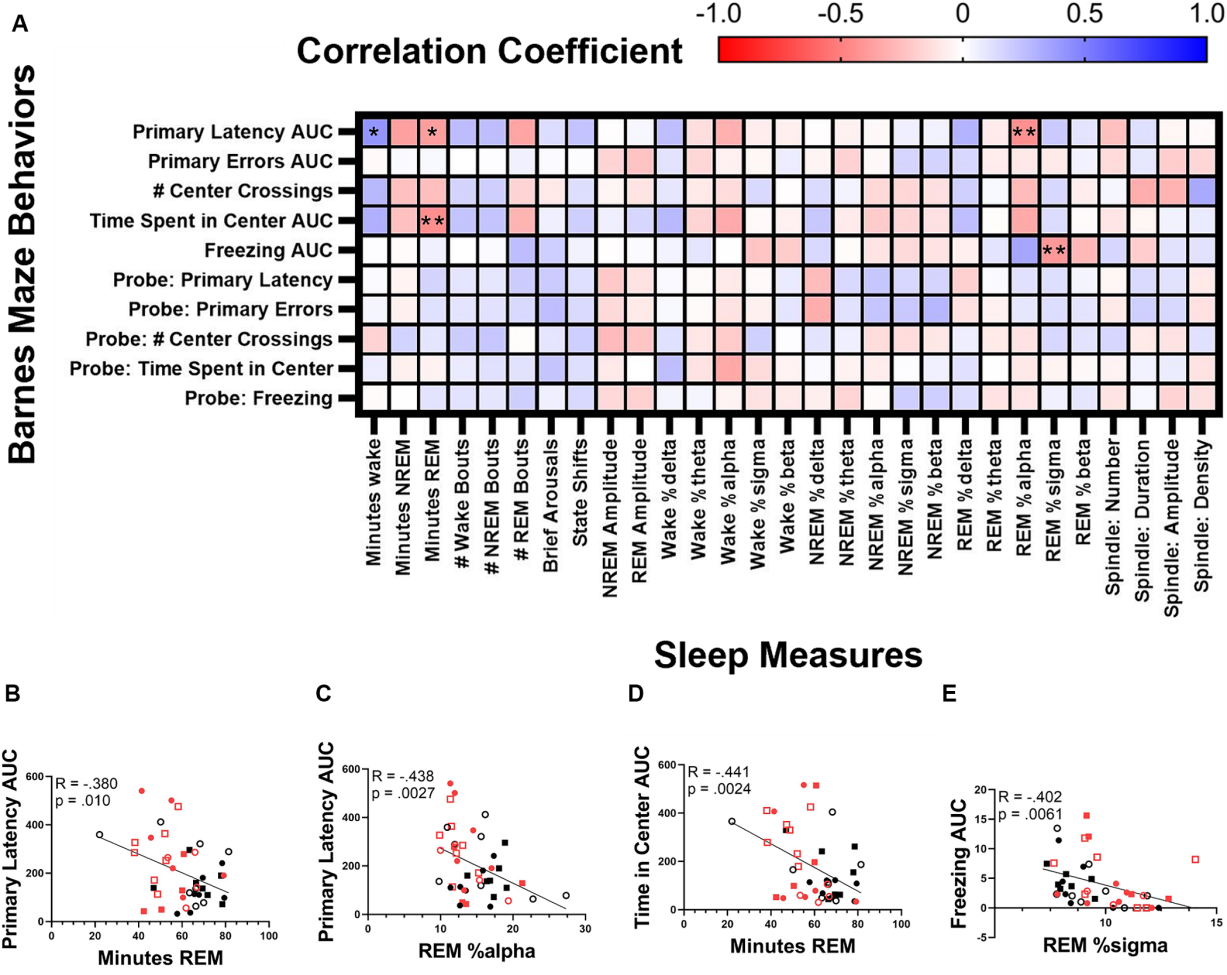
Correlation between sleep and cognitive measures. Correlation matrix of cognitive measures collected during the Barnes maze test and sleep measures **(A)**. Correlation of the area under the curve of primary latency and minutes of REM **(B)** and relative REM alpha power **(C)**. Correlation of the time spent in center and the minutes of REM **(D)**. Correlation between time spent in freezing behavior and REM sigma power **(E)**. Statistical significance was calculated by two-tailed Pearson’s correlation (with Bonferroni correction).

Finally, we correlated sleep and cognitive measures (Figure 9A). We found that primary latency was inversely correlated with both total REM time (Bonferroni corrected *p* = 0.010, Pearson R = −0.380; Figure 9B) and REM alpha power (Bonferroni corrected *p* = 0.0027, Pearson R = −0.438; Figure 9C). Time spent in the center of the testing table was also inversely correlated with total REM time (Bonferroni corrected *p* = 0.0024, Pearson R = −0.441; Figure 9D) and freezing behavior correlated inversely with REM sigma power (Bonferroni corrected *p* = 0.0061, Pearson R = −0.402; Figure 9E).

## 4 Discussion

Alzheimer’s disease is a very serious neurodegenerative disease for which there is no known cure. Therapeutic strategies for halting or reversing the progression of disease pathology have been areas of immense research interest but have proved elusive (96). As such, it is possible that intervention during the prodromal or early disease stages may prove to be most effective. One candidate for intervention is sleep due to the fact that sleep disruptions in AD patients often present years before the onset of cognitive symptoms (97). As disrupted sleep is linked to acceleration of AD pathology and also cognitive deficits, enhancing sleep lead to slower disease progression as well as cognitive enhancements. In fact, modulating light levels, has been shown to decrease cortical Aβ42 levels in 5xFAD mice (76). In this study, we explored the possibility of manipulating sleep via a circadian-focused intervention: feeding time. If a controlled feeding schedule can enhance sleep in AD patients leading to disease or symptom management, it would be inexpensive and easy to implement.

With this study, we intended to evaluate both female and male 5xFAD mice, as many studies are done solely on male mice. Importantly, AD affects women and men differently. Women are 2–3 times more likely to be affected, present differently in a clinical setting, and have reacted differently to many treatments (98). Furthermore, the 5xFAD genotype has been shown to affect female and male mice differently (74). We also wanted to evaluate the effects of correct and incorrect phase-restricted feeding in 5xFAD and WT mice to determine if the 5xFAD genotype modulates the response to feeding time. Perhaps the most applicable of our aims was to determine if we can modulate sleep and cognition via feeding time.

Generally in phase-restricted feeding studies, the eating window is short, between 4 and 10 h, and is tested only during the “correct” time window, i.e., during the dark phase for mice. Consequently it is not possible to separate the effects of a limited time duration of food availability from the effects of the time of day that food availability occurs. The objective of the present study was to examine the role of the time of day, or phase of the light–dark cycle, when food consumption occurs while keeping the time window fixed at 12 h. We thus compared “incorrectly” timed feeding (light-fed) with “correctly” timed feeding (dark-fed) in order to differentiate the effects of feeding time.

Previous studies have demonstrated improved cognitive performance with phase-restricted feeding in mouse models of AD (73, 77, 78). Alterations in sleep/wake or activity/rest patterns have been reported in response to phase-restricted feeding. We sought to extend those findings to examine not only the circadian impact of a meal-time Zeitgeber, but whether characteristics of sleep EEG associated with learning and memory function could be affected by a circadian behavior-focused intervention. Our ability to collect and analyze cognitive behavior and EEG/EMG data to determine characteristics of wake, NREM, and REM has allowed us to test the potential role played by sleep in mediating the effects of circadian feed/fast cycles on cognitive function.

Phase-restricted feeding did not appear to adversely affect the health of the mice; all mice maintained an appropriate weight throughout the study. Previous studies in male mice have reported that male mice gain more weight when fed during the 12-h light phase than when fed the same amount over the 12-h dark phase (35). Our data recapitulates this finding, despite the fact that the mice in this experiment began phase-restricted feeding at ~3.5 months instead of at 9 weeks. In male mice, the 5xFAD genotype has no impact on body weight change. These results are in contrast to those collected from female mice where the opposite effects were seen. In female mice, the 5xFAD genotype and dark phase feeding caused increased weight gain. We did not collect data on the calorie intake in any of the mice so we do not know if females eat less on an alternative feeding schedule.

We wanted to investigate wake and NREM independently of REM due to the differences in homeostatic and circadian control of the two sleep states. We first wanted to characterize wake and NREM in all groups. In our study, 5xFAD mice spend more time awake and less time asleep and have fewer wake and NREM bouts than WT mice. In WT mice, incorrectly-timed feeding decreases NREM and causes a disruption of the wake and NREM bout and brief arousal rhythms, especially in males. In 5xFAD mice, sex and feeding time interact, resulting in female 5xFAD mice increasing and male 5xFAD mice decreasing wake in response to incorrectly-timed feeding. In all of these measures, 5xFAD mice are less sensitive to feeding time than WT mice. Despite the changes outlined, genotype did not impact the EEG power spectra in wake or NREM.

Next, we looked at the circadian aspects of NREM in all groups. Here, we report that feeding time in WT mice modulates the timing of NREM. Again, we see that this effect is diminished in 5xFAD mice. In other measures of rhythm such as the proportion of wake and NREM which occur in the correct phase and state amplitude, both WT and 5xFAD mice decrease rhythm strength in response to feeding during the light phase. This result was predicted, as incorrectly-timed feeding was hypothesized to disrupt vigilance state timing and rhythm. Interestingly, 5xFAD mice have an increased proportion NREM in the light phase as well as increased NREM rhythm amplitude. This result was unexpected as we had predicted that the genotype would disrupt vigilance state rhythms in a similar manner. Instead, the genotype results in decreased NREM in the dark phase which enhances these measures. Interestingly, this phenomenon has also been reported also in the APP23 mouse model of AD (99). There has been little research on NREM deprivation over the dark phase. It is; however, reasonable to assume that this sleep which is consistently seen in healthy mice is beneficial to the mouse.

We reviewed many of the same measures in REM. In many ways, the patterns in REM reflect the results of NREM. 5xFAD mice had less REM overall, which was concentrated in the light phase. Also, REM bout number and minutes of REM over the day were less tractable in response to feeding time in 5xFAD than WT mice. Incorrectly-timed feeding reduced the amount of REM in the light phase as well as REM amplitude.

To the best knowledge of the authors, there have been no previously published studies which report PSD data in 5xFAD mice or in mice which have undergone phase-restricted feeding at different circadian times. Consistent with our previous findings, feeding time produces changes in the wake and REM PSD of WT mice that are not reproduced in 5xFAD mice. Furthermore, females are more sensitive to light-time feeding than males.

Finally, we characterized sleep spindles in WT and 5xFAD mice. Sleep “spindles” or sigma power bursts during stage 2 Non-REM are associated with learning and memory formation (100). Unlike the other sleep measures, feeding phase did not affect sleep spindle characteristics during undisturbed conditions. 5xFAD mice had fewer sleep spindles but they were slightly longer. Sleep spindles did not correlate with Barnes maze performance but our recording of sleep spindles occurred before the learning challenge and therefore represent a baseline, not post-learning environment. Sleep spindles have not previously been characterized in the 5xFAD mouse model but a decrease in the number of sleep spindles have been reported in Alzheimer’s disease patients (101).

Overall, our results indicate that sleep in 5xFAD mice is less affected by feeding time than WT mice. This may be due to a heightened response to photic cues in AD model mice which are shown to re-entrain to light cycles more quickly (102). Alternatively, the diminished effect of feeding time in 5xFAD mice may reflect an impairment in the normal circadian entrainment response to meal time. This suggests that AD-associated disturbances in circadian timing affects not only the timing of behavioral rhythms but also the feedback effects of these rhythms.

Throughout the study, we consistently noted the impact of sex in that females were more significantly affected by both the 5xFAD genotype as well as feeding time. Sex differences in the 5xFAD model have been demonstrated previously which align with these findings. When compared to males, female 5xFAD mice have been found to have higher levels of Aβ deposition (103–106), hippocampal proteome changes (106), reduced brain glucose metabolism (103), and increased markers for inflammation (104, 105). These pathology differences align with previous findings that female mice are more affected by the 5xFAD genotype. In a previous study, Female 5xFAD mice had 12% less total sleep than WT mice while male mice showed no deficiencies (74). In the same study, both male and female 5xFAD mice experienced fragmented sleep, although female sleep was more fragmented than males (74). Beyond sleep, female mice experience increased deficits in learning and exploration (105, 107). Additionally, the metabolic consequences of high-fat diet in AD model mice produce a higher increase of Aβ plaques in female mice (108).

In a Barnes maze test of spatial learning, all groups of mice learned over the course of the test as measured by primary latency and primary errors. In females, 5xFAD mice have a longer primary latency and more primary errors. This is mitigated by feeding restricted to the dark phase but these effects are not seen in male mice. In a previous study during a test of hippocampal working memory, female and male 5xFAD mice did not demonstrate deficits compared to WT mice but, interestingly, the directionality in change between 6 and 12 months was opposite in female and male mice, consistent with the results presented here (109). Behavioral differences have been seen in 5xFAD mice which may confound cognitive tests. Both female and male 5xFAD mice are hyperactive when compared to WT mice measured at 6 and 12 months (109). In an open field test, 5xFAD mice prefer the center as compared to WT mice at 8 months (110). This is consistent with our finding in female mice that the 5xFAD genotype causes mice to spend more time in the center of the maze table although we did not observe the same result in male mice. Consistently, 5xFAD also mice demonstrate decreased anxiety as indicated by the elevated plus maze (110).

Following the cognitive tests, we correlated sleep and cognitive measures. REM amount correlates positively with spatial learning and decreased anxiety behavior. REM alpha power also correlates positively with spatial learning although there is a paucity of research on REM alpha power and learning in mice. The freezing behavior was quantified and represented a very small amount of time but did correlate with REM sigma power. REM sigma power has previously been correlated with fear behavior in rats (111).

## 5 Limitations

We have not reported here pathology data but these 5xFAD mice would have already developed a significant plaque burden before phase-restricted feeding was begun. Importantly, although amyloid plaque formation is an important hallmark in the development of AD, plaque load is not well correlated with cognitive performance (112, 113). It would be worthwhile to compare pathology in 5xFAD mice which are begun on a phase-restricted feeding, or other sleep enhancement protocol, at different stages of disease progression and for varying lengths of time.

One important factor to consider in this study is that we were unable to accurately measure the amount of food consumed due to the crumbly consistency of the high-fat chow. This did render us unable to determine rate of gain differences between food access restricted to different phases. It also precluded us from drawing conclusions from the effects of calorie consumption on the metabolic effects of incorrectly-timed feeding. We did; however, monitor body weight throughout the experiment and all mice maintained an appropriate weight throughout. It is unlikely that the results seen here are due to differences in the amount of calories consumed.

Lastly, it is necessary to note that the timing cues may come from mouse handling (114). Our mice were handled regularly, twice a day near lights on/off which may have provided additional environmental zeitgebers.

## 6 Conclusion

Our study determined that the 5xFAD genotype *per se* results in the disruption of vigilance states, specifically in a loss of NREM and REM. These vigilance states are less affected by feeding time in 5xFAD mice when compared to WT mice although, the mechanism behind this is unknown, and female mice are more affected than male. Our results also indicate that the timing of the feeding window is important for vigilance state characteristics, even if the length of the window is maintained. We also demonstrate that, in female mice, restricting feeding time to the dark phase in 5xFAD mice ameliorates some on the cognitive deficits. REM amount correlates positively with spatial learning and decreased anxiety behavior; the more REM a mouse got over 24 h, the faster they found the target hole and the less time they spent in the center of the testing table. These findings are consistent with the role of REM in memory (14).

## Data availability statement

The raw data supporting the conclusions of this article will be made available by the authors, without undue reservation.

## Ethics statement

The animal study was approved by Northwestern University Institutional Animal Care and Use Committee. The study was conducted in accordance with the local legislation and institutional requirements.

## Author contributions

KC: Formal analysis, Investigation, Methodology, Supervision, Visualization, Writing – original draft, Writing – review & editing. PJ: Conceptualization, Funding acquisition, Investigation, Methodology, Supervision, Writing – review & editing. CO: Formal analysis, Methodology, Supervision, Visualization, Writing – review & editing. XL: Formal analysis, Methodology, Supervision, Writing – review & editing. SK: Formal analysis, Methodology, Supervision, Writing – review & editing. CL: Formal analysis, Methodology, Supervision, Writing – review & editing. ES: Formal analysis, Methodology, Supervision, Writing – review & editing. FT: Conceptualization, Funding acquisition, Project administration, Supervision, Writing – review & editing. MV: Conceptualization, Formal analysis, Funding acquisition, Investigation, Methodology, Project administration, Supervision, Visualization, Writing – original draft, Writing – review & editing.

## Supplementary material

SUPPLEMENTARY FIGURE 1 NREM following cage switch. NREM latency **(A)** after ZT0 in all experimental groups as well as control mice which did not undergo cage-switching.

SUPPLEMENTARY FIGURE 2 Body weight change and vigilance state amounts. The percentage of time spent in wake **(A)**, NREM **(B)**, and REM **(C)** were correlated with the percentage change in body weight from baseline. Data are presented as individual values. Statistical significance was determined by a two-tailed Pearson correlation. *N* = 3–7 mice per group.

SUPPLEMENTARY FIGURE 3 NREM power spectral density. The NREM power spectral density in female **(A,C,E,G)** and male **(B,D,F,H)** mice. The PSDs were combined first by genotype: WT **(A,B)** and 5xFAD **(C,D)** and then by feeding time: dark **(E,F)** and light **(G,H)**. Data are presented as mean ± SEM. Statistical significance was determined by 2-way ANOVA (frequency × feeding time; frequency × genotype) and 3-way ANOVA (sex × genotype × frequency; feeding time × genotype × frequency). ^**^*p* < 0.01; ^***^*p* < 0 .001; ^****^*p* < 0.0001. *N* = 3–7 mice per group.

SUPPLEMENTARY FIGURE 4 Barnes maze probe test. The primary latency **(A)** and primary errors **(B)** from the Barnes maze probe test and the correlation of the probe data with the training data [primary latency **(C)**, primary errors **(D)**].
