# Supplementary figures and images for: The impacts of sex and the 5xFAD model of Alzheimer’s disease on the sleep and spatial learning responses to feeding time
Source: Front Neurol. 2024 Jul 31;15:1430989. doi: 10.3389/fneur.2024.1430989 (PMC11322461; doi:10.3389/fneur.2024.1430989)

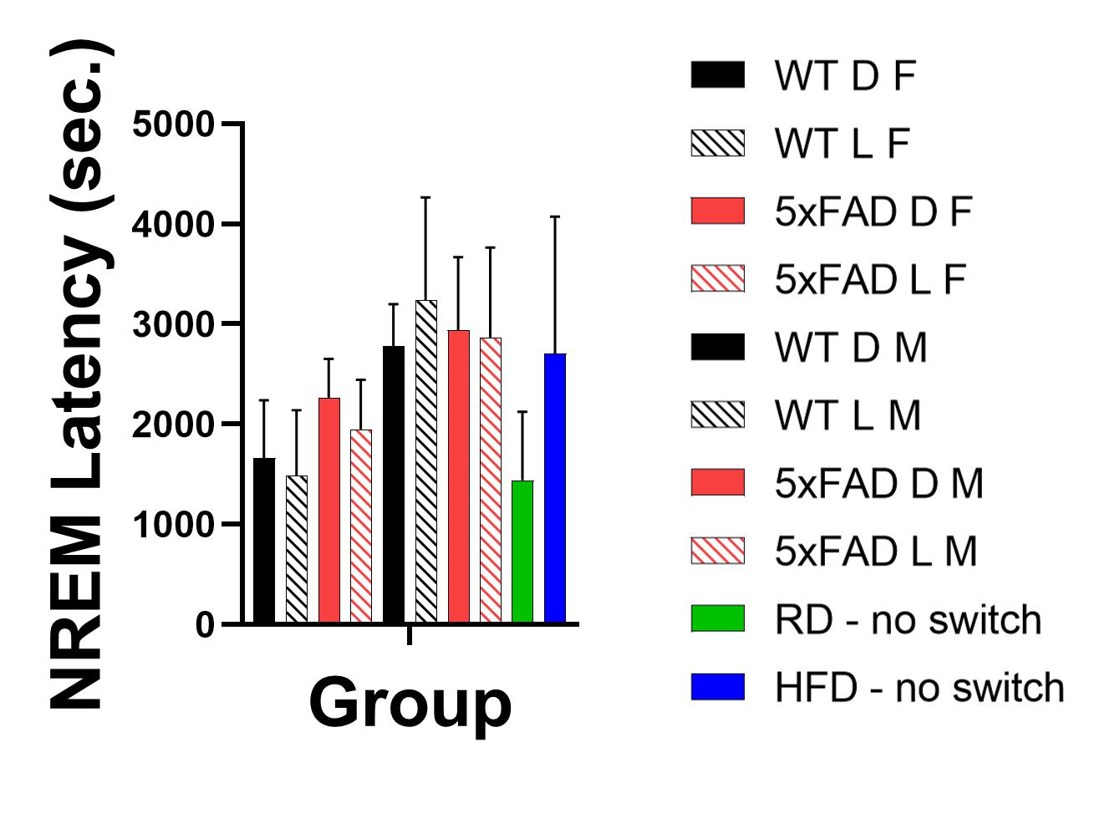

Supplement: SUPPLEMENTARY FIGURE 1 — NREM following cage switch. NREM latency (A) after ZT0 in all experimental groups as well as control mice which did not undergo cage-switching. [file Image_1.TIF]

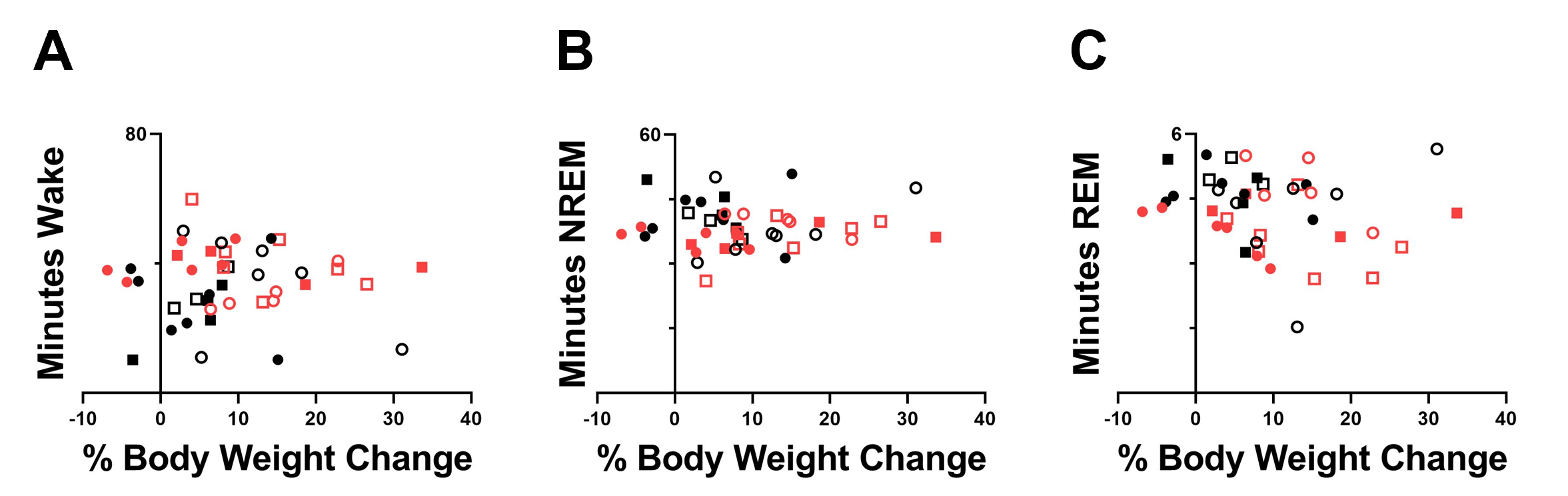

Supplement: SUPPLEMENTARY FIGURE 2 — Body weight change and vigilance state amounts. The percentage of time spent in wake (A), NREM (B), and REM (C) were correlated with the percentage change in body weight from baseline. Data are presented as individual values. Statistical significance was determined by a two-tailed Pearson correlation. N = 3–7 mice per group. [file Image_2.TIF]

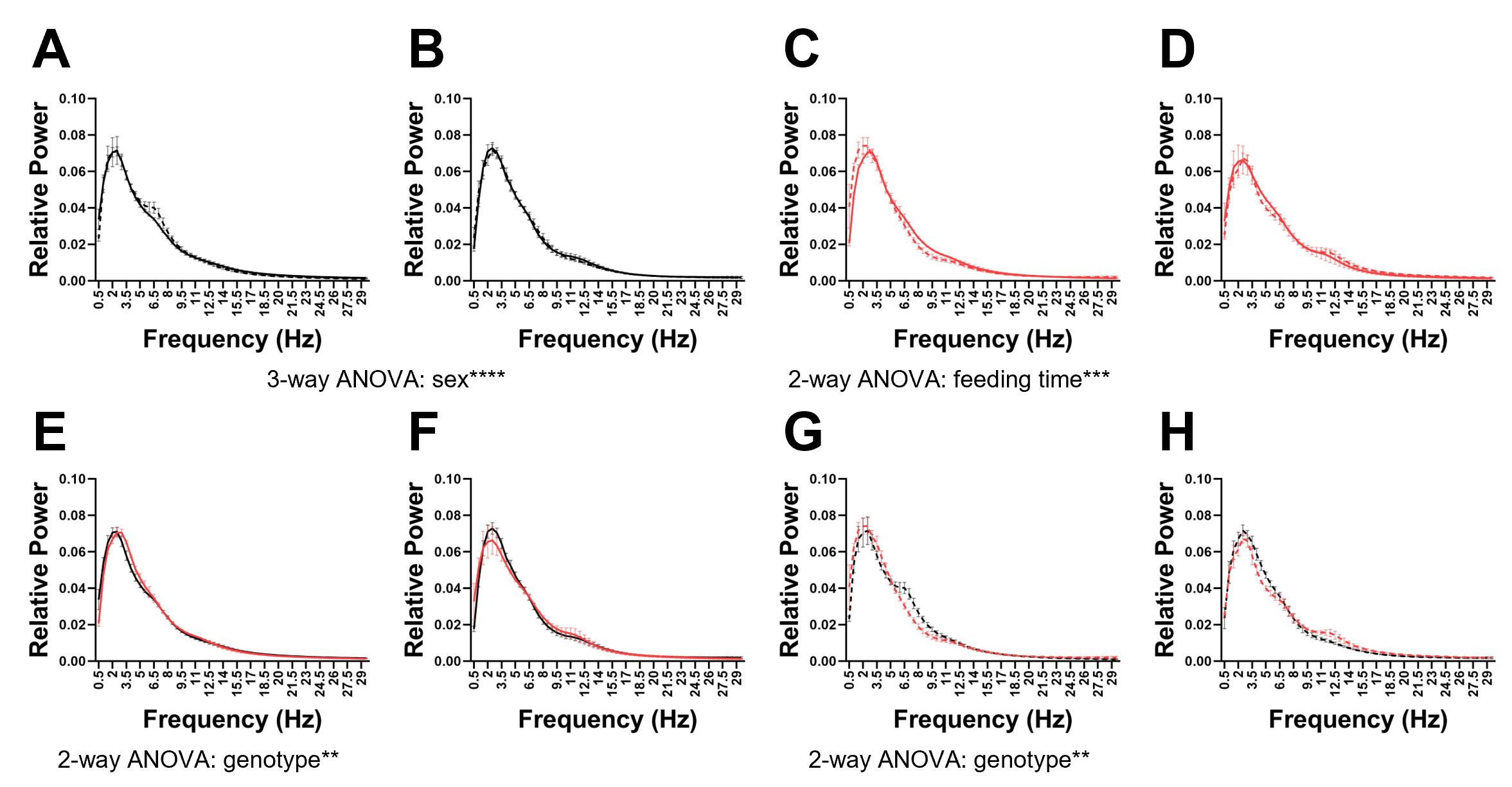

Supplement: SUPPLEMENTARY FIGURE 3 — NREM power spectral density. The NREM power spectral density in female (A,C,E,G) and male (B,D,F,H) mice. The PSDs were combined first by genotype: WT (A,B) and 5xFAD (C,D) and then by feeding time: dark (E,F) and light (G,H). Data are presented as mean ± SEM. Statistical significance was determined by 2-way ANOVA (frequency × feeding time; frequency × genotype) and 3-way ANOVA (sex × genotype × frequency; feeding time × genotype × frequency). **p < 0.01; ***p < 0 .001; ****p < 0.0001. N = 3–7 mice per group. [file Image_3.TIF]

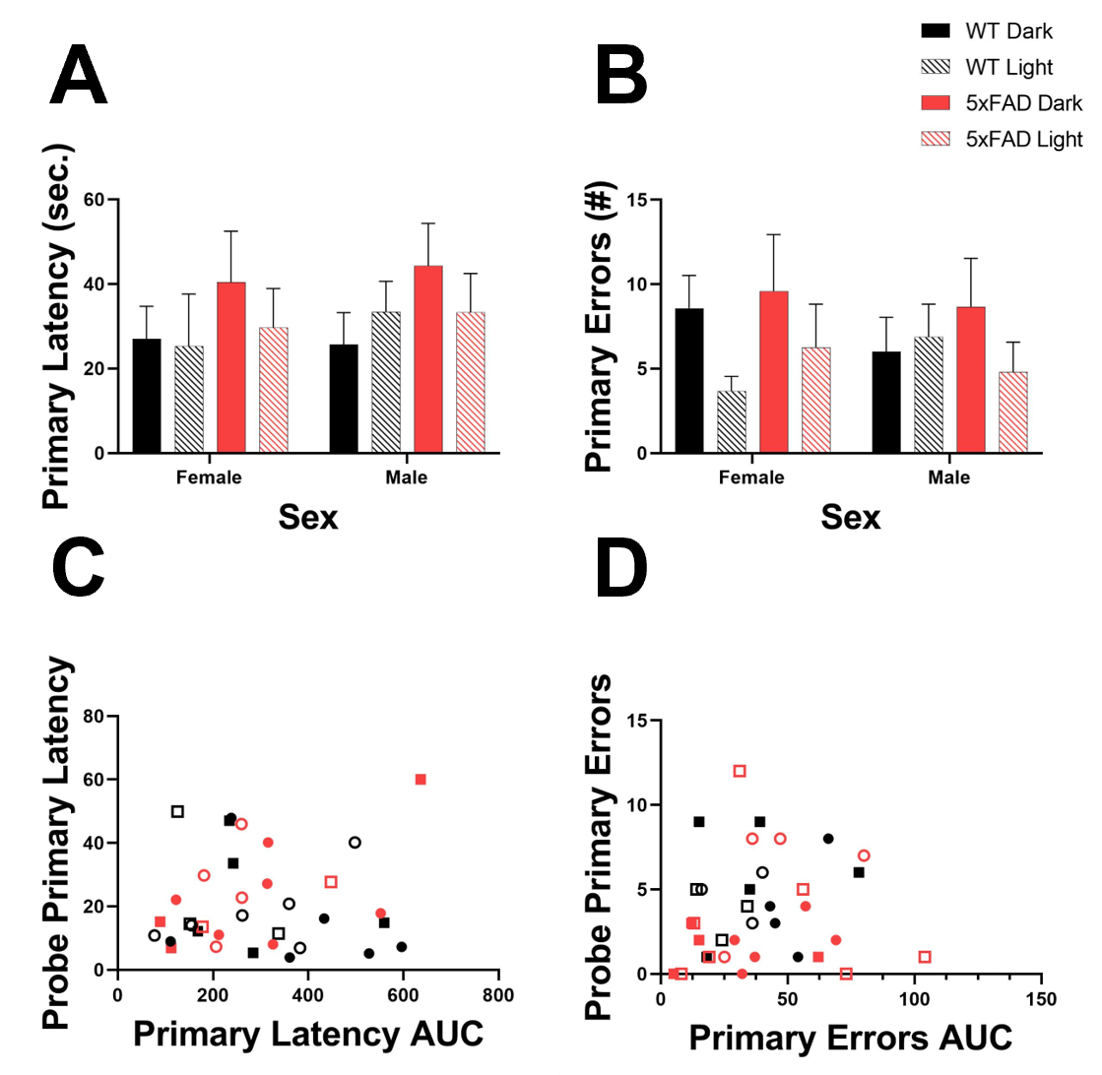

Supplement: SUPPLEMENTARY FIGURE 4 — Barnes maze probe test. The primary latency (A) and primary errors (B) from the Barnes maze probe test and the correlation of the probe data with the training data [primary latency (C), primary errors (D)]. [file Image_4.TIF]
